# RETRACTION: EZH2 Promotes Invasion and Tumour Glycolysis by Regulating STAT3 and FoxO1 Signalling in Human OSCC Cells

**DOI:** 10.1111/jcmm.71083

**Published:** 2026-03-04

## Abstract

**RETRACTION**: ZhengM. , CaoM.‐X. , LuoX.‐J. , LiL. , WangK. , WangS.‐S. , WangH.‐F. , TangY.‐J. , TangY.‐L. , LiangX.‐H. , “EZH2 Promotes Invasion and Tumour Glycolysis by Regulating STAT3 and FoxO1 Signalling in Human OSCC Cells,” Journal of Cellular and Molecular Medicine 23, no. 10 (2019): 6942–6954, 10.1111/jcmm.14579.31368152 PMC6787444

The above article, published online on 31 July 2019 in Wiley Online Library (wileyonlinelibrary.com) has been retracted by agreement between the journal Editor‐in‐Chief, Stefan N. Constantinescu; The Foundation for Cellular and Molecular Medicine; and John Wiley and Sons Ltd. The retraction has been agreed due to concerns raised by third parties. Specifically, instances of image duplication have been identified between Figures 2C and 3C, between Figures 2A and 3A, and between Figures 2D and 5A. Furthermore, image elements in Figures 2C and 3C have been published previously by the same author group in a different scientific context. The clarification provided by the authors was insufficient to adequately address the concerns raised, as additional inaccuracies were identified by the authors during their review of the original data. Accordingly, the article has been retracted, as the editors no longer have confidence in the overall reliability of the data or in the conclusions presented in the article. The authors have been informed of the retraction decision but were not available for final confirmation.

**RETRACTION**: M. Zheng , M.‐X. Cao , X.‐J. Luo , L. Li , K. Wang , S.‐S. Wang , H.‐F. Wang , Y.‐J. Tang , Y.‐L. Tang , X.‐H. Liang , “EZH2 Promotes Invasion and Tumour Glycolysis by Regulating STAT3 and FoxO1 Signalling in Human OSCC Cells,” Journal of Cellular and Molecular Medicine 23, no. 10 (2019): 6942–6954, 10.1111/jcmm.14579.31368152 PMC6787444

The above article, published online on 31 July 2019 in Wiley Online Library (wileyonlinelibrary.com) has been retracted by agreement between the journal Editor‐in‐Chief, Stefan N. Constantinescu; The Foundation for Cellular and Molecular Medicine; and John Wiley and Sons Ltd. The retraction has been agreed due to concerns raised by third parties. Specifically, instances of image duplication have been identified between Figures 2C and 3C, between Figures 2A and 3A, and between Figures 2D and 5A. Furthermore, image elements in Figures 2C and 3C have been published previously by the same author group in a different scientific context. The clarification provided by the authors was insufficient to adequately address the concerns raised, as additional inaccuracies were identified by the authors during their review of the original data. Accordingly, the article has been retracted, as the editors no longer have confidence in the overall reliability of the data or in the conclusions presented in the article. The authors have been informed of the retraction decision but were not available for final confirmation.

